# Direct identification of HLA class I and class II-restricted T cell epitopes in pancreatic cancer tissues by mass spectrometry

**DOI:** 10.1186/s13045-022-01373-6

**Published:** 2022-10-25

**Authors:** Kenji Fujiwara, Yingkuan Shao, Nan Niu, Tengyi Zhang, Brian Herbst, Mackenzie Henderson, Stephen Muth, Pingbo Zhang, Lei Zheng

**Affiliations:** 1 Department of Oncology, Johns Hopkins University School of Medicine, Baltimore, MD 21287 USA; 2 The Sidney Kimmel Cancer Center, Johns Hopkins University School of Medicine, Baltimore, MD 21287 USA; 3 The Pancreatic Cancer Precision Medicine Center of Excellence Program, Johns Hopkins University School of Medicine, Baltimore, MD 21287 USA; 4 The Cellular and Molecular Medicine Graduate Program, Johns Hopkins University School of Medicine, Baltimore, MD 21287 USA; 5 Department of Surgery, Johns Hopkins University School of Medicine, Baltimore, MD 21287 USA; 6 Department of Ophthalmology, Johns Hopkins University School of Medicine, Baltimore, MD 21287 USA; 7 Department of Surgery, Kimura Hospital, Fukuoka, Japan; 8 The Second Affiliated Hospital, Zhejiang University School of Medicine, Hangzhou, China

**Keywords:** Pancreatic ductal adenocarcinoma, Mass spectrometry, T cell epitopes, Peptidome analysis, Immunotherapy

## Abstract

**Background:**

Identifying T cell epitopes on pancreatic ductal adenocarcinoma (PDAC) associated antigens or neoantigens has been a challenge. In this study, we attempted to identify PDAC T cell epitopes by mass spectrometry (MS).

**Methods:**

We isolated HLA class I (HLA-I) and HLA class II (HLA-II)-restricted peptides, respectively, from tissues of human PDAC by using the pan-HLA-I or pan-HLA-II affinity purification column and identified T cell epitopes by peptidome analysis with MS.

**Results:**

Through peptidome analysis, we identified T cell epitopes shared by multiple patients with different HLA types and those containing sequences of both anti-HLA-I and HLA-II antibodies-affinity purified peptides. The identified epitopes bound non-matched HLA molecules and induced T cell response in peripheral T cells from both HLA-type matched and non-matched patients. Peptides containing both HLA class I and class II epitopes were able to induce polyfunctional cytokine responses in peripheral T cells.

**Conclusions:**

T cell epitopes in PDAC can be discovered by the MS approach and can be designed into vaccine and TCR-T cell therapies for both HLA-type matched and non-matched patients.

**Supplementary Information:**

The online version contains supplementary material available at 10.1186/s13045-022-01373-6.

To the editor

It was a challenge to identify T cell epitopes in pancreatic ductal adenocarcinoma (PDAC) largely due to lack of knowledge on immunodominant antigens [1] and effective technical approaches. Although in silico epitope prediction from whole-exome sequencing results has been used to predict mutation-associated neoepitopes [2–4], such an approach may not predict high-affinity T cell-receptor binding epitopes if the tumors have low tumor mutation burdens (TMB) [5]. In this study, we isolated HLA class I(HLA-I) and HLA class II(HLA-II)-restricted peptides, respectively, from tissues of human PDAC, a low-TMB tumor, by using the pan-HLA-I or pan-HLA-II affinity purification column and identified T cell epitopes by peptidome analysis with mass spectrometry (MS). Bioinformatics analysis identified 553 and 1709 HLA-I bound peptides from two human PDAC cell lines, Panc10.05 and Panc6.03, respectively (Fig. 1A), and similar numbers of HLA-I bound peptides from 12 surgically resected human PDAC tissues (Additional file 1: Table S1, Additional file 2: Table S2, Fig. 1B–C, Additional file 1: Fig. S1). The numbers of peptides with different lengths peaked at 9 amino-acid, an anticipated length of HLA-I bound peptides [6, 7]. We filtered 9-mer peptides from 8 PDAC specimens whose HLA types were available and predicted their binding affinity to their corresponding HLA-I types by using NetMHC-4.0 [7]. However, the results suggests that the predicting algorithm may have missed many HLA-binding peptides (Fig. 1D, Additional file 1: Fig. S2). Interestingly, we found that eluted epitopes were shared among different PDACs as well as PDAC cell lines (Additional file 1: Table S3, Fig. S3–4). For further validation, we chose eight shared peptides (Fig. 1E), which were among predicted high-affinity binding peptides shared by multiple patients (Fig. 1F).

**Fig. 1:**
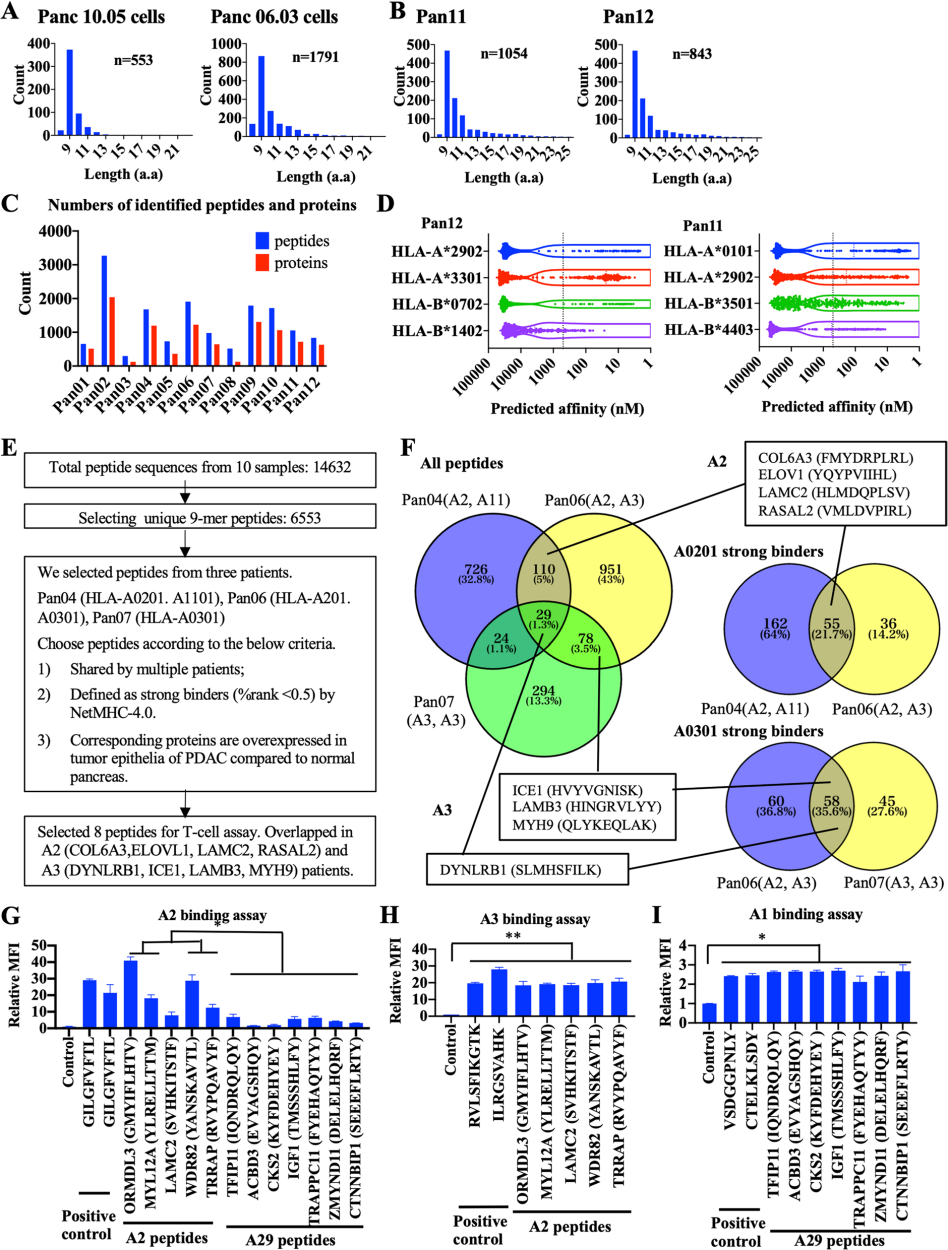
Mass spectrometry analysis of HLA Class I epitopes in PDAC tumor cell lines and tissues. MaxQuant was used to identify the peptide sequences with a false discovery rate (FDR) of 1%. **A** Histograms show the numbers of different lengths of peptides affinity purified by anti-HLA Class I antibody from human PDAC cell lines, Panc10.05 and Panc06.03. These peptides correspond to 363 and 1238 unique proteins, respectively. **B** Representative histograms show the numbers of different lengths of peptides affinity purified by anti-HLA Class I antibody from human PDAC tissues. **C** The numbers of HLA Class I epitopes and their associated proteins identified from each individual PDAC tissues. From the 10 PDACs, a total of 14,632 peptides and 11,849 unique peptides, corresponding to 6086 non-redundant proteins, were identified. Note that the numbers of eluted peptides from different PDAC specimens varied between 296 and 3270 (1331 on average). These peptides correspond to 123–2041 proteins (782 on average), respectively. **D** Predicted HLA Class I binding affinity of eluted peptides from representative PDAC tissues, Pan12 and Pan11, using the NetMHC4.0 algorithm. The black dot lines represent the 500 nM threshold of high binding affinity. Note that 339 eluted peptides and 219 eluted peptides from the Pan12 PDAC specimen (81.7% and 52.8% of the total of 415 9-mer peptides, respectively) showed a low predicted binding affinity to the patient’s class I HLA types, HLA-A*2902 and HLA-A*3301, respectively. Similarly, 343 eluted peptides and 319 eluted peptides from the Pan11 PDAC specimen (73.3% and 68.2% of the total of 468 9-mer peptides, respectively) showed a low predicted binding affinity to HLA-A*0101 and HLA-A*2902, respectively. A similar degree of netMHC in missing the peptides with high binding affinity was reported in the prior study [11]. **E** Criteria of selecting peptides for validation: (1) those that are shared by multiple patients; (2) those whose predicted HLA-binding affinity ranks among the top 0.5% of all peptides, which is the recommended threshold for the selection of peptides by NetMHC); (3) those whose corresponding proteins are overexpressed in tumor epithelia of PDAC compared to normal pancreas according to the Human Protein Atlas (https://www.proteinatlas.org/). These criteria were used with a consideration of developing therapeutic agents in the future. Eight peptides that met the selection criteria include four HLA-A2 peptides (COL6A3, ELOVL1, LAMC2, RASAL2) and four HLA-A3 peptides (DYNLRB1, ICE1, LAMB3, MYH9) (Additional file 1: Fig. S4). **F** Numbers of HLA class I peptides from representative PDAC samples including Pan04, Pan06, and Pan07 and those of completely overlapped peptides among all three or any two of three PDAC samples were indicated (left). Numbers of peptides considered as strong binders (ranks among the top 0.5%) for HLA-A0201 (upper right) and HLA-A0301 (lower right) in Pan06 and Pan07, respectively, and those of overlapped peptides between Pan06 and Pan07 were also indicated. The sources of eight selected peptides were indicated. **G**–**I** T2 cell binding assays of selected HLA-A2 and A29 peptides binding to HLA-A2 expressing T2 cells (**G**), HLA-A3 expressing T2 cells (**H**), and HLA-A1 expressing T2 cells (**I**). Twelve peptides that consisted of five peptides (ORMDL3, MYL12A, LAMC2, WDR82, TRRAP) shared by multiple HLA-A2 PDACs and seven peptides (TFIP11, ACBD3, CKS2, IGF1, TRAPPC11, ZMYND11, CTNNBIP1) shared by multiple HLA-A29 PDACs were selected according to the criteria listed in (**E**) (Additional file 1: Fig. S8) and their sequences were shown. Controls indicate negative control peptides. MFI: mean fluorescent intensity. Unpaired *t* test and 1-way ANOVA was used for comparing between samples. *p < 0.05, **p < 0.01

Surprisingly, T cell response as demonstrated by the expression of either IFN-*γ* or granzyme B or both was significantly stimulated by these peptides not only in the PBMC from at least one of two patients whose tumors were used for identifying these peptides, but also in those from HLA-type unmatched patients (Additional file 1: Fig. S5–7). Thus, we selected five peptides shared by multiple HLA-A2 PDACs and seven peptides shared by multiple HLA-A29 PDACs (Additional file 1: Fig. S8) and examined their binding to HLA molecules in the T2-binding assay (Fig. 1G). The results showed that the peptides have a specific binding to HLA-A2 and A29, respectively, however, five HLA-A2 peptides also bound to HLA-A3 (*p* < 0.01, Fig. 1H) and seven HLA-A29 peptides bound to HLA-A1 (*p* < 0.05, Fig. 1I). Note that T2 cells used were not reported to bind A2 peptides and A29 peptides, respectively [8]. These peptides are not predicted to bind HLA-A1 or A3 according to NetMHC (Additional file 1: Table S4), either. Moreover, the A29 peptides and the A2 peptides were able to stimulate T cells from an HLA-(A2, A11) patient and an HLA-(A29, A33) patient, respectively (Additional file 1: Fig. S9–10).

After we used the pan-HLA class I affinity purification column to bind the HLA-I peptides in the lysate of PDAC tissues, we subjected the flow-through for HLA-II peptide isolation with the pan-HLA-II affinity purification column. The numbers of HLA-II peptides with different lengths peaked at 14–16 amino acids (Fig. 2A, Additional file 1: Fig. S11). We found that HLA-II and HLA-I epitopes purified separately from the same PDAC specimens frequently contained overlapped peptide sequences (Fig. 2B). Next, we selected 30 peptides shared between two PDACs and eluted from both HLA-I and HLA-II affinity purification columns, respectively, and according to similar criteria in Fig. 1E, narrowed them down to 6 peptides whose binding to HLA-II could be predicted by NetMHCIIpan-4.0 and NetMHC-4.0 according to the patients’ known HLA-II types [9]. We then synthesized 12–21-mer peptides according to the core MHC binding sequences that were predicted by NetMHCIIpan (Additional file 1: Table S5) and examined the ability of these synthetic peptides in stimulating the IFN-*γ*, TNF-*α*, and IL-2 expression from T cells in HLA-type unmatched PBMCs (Fig. 2C). The results showed that some peptides could stimulate the expression of all three cytokines, suggesting that polyfunctional T cells are induced by these peptides.

**Fig. 2:**
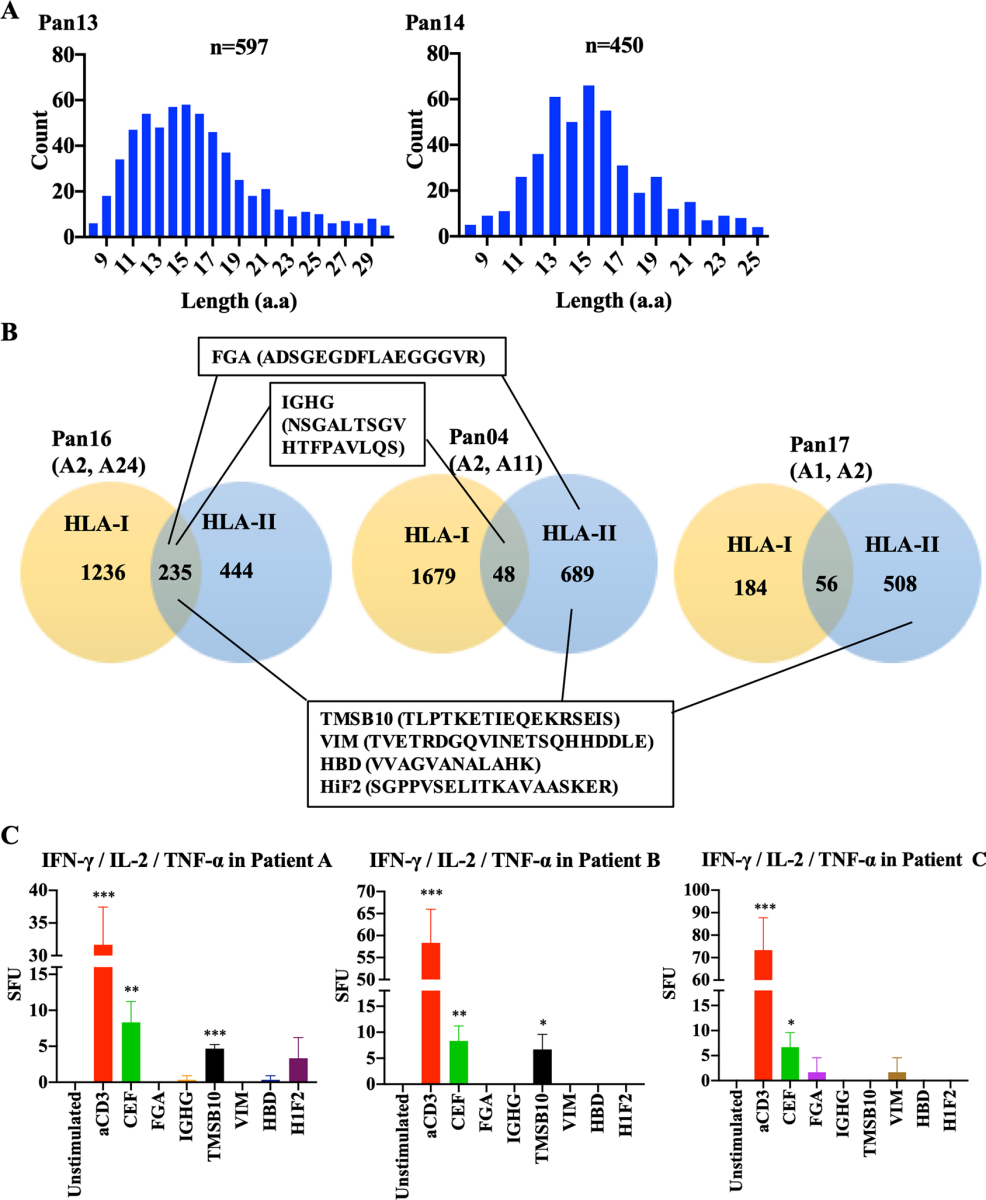
Mass spectrometry analysis of HLA Class II epitopes in PDAC tumor tissues. MaxQuant was used to identify the peptide sequences. The MS analysis of eluted HLA-II peptides showed an average of 490 peptide sequences (ranging between 249 and 689), corresponding to an average of 116 proteins (ranging between 62 and142) from six PDAC tissue samples. **A** Histograms show the numbers of different lengths of peptides affinity purified by anti-HLA Class II antibody from two representative human PDAC tissue samples, Pan13 and Pan14. **B** Numbers of total HLA class I peptides, HLA class II peptides, and completely overlapped peptides between HLA-I and HLA-II peptides were indicated. Three representative PDAC samples were shown. The sources of six selected peptides were indicated. **C** Ability of selected, HLA class I/II-overlapped peptides in stimulating single cells to express IFN-y, IL-2 and TNF-a in FluoroSpot assays was shown in the histograms. PBMC samples from three representative patients were shown. Spot forming unit (SFU) is the number of spots per 10^6^ PBMCs. Shown is SFU of each peptide after subtracting that of a negative control peptide; and error bars represent the percentages of deviation. “Unstimulated” indicates the reaction in absence of peptides. If the SFU of a peptide in a sample is less than that of the negative control peptide, it is set as zero; and such a result would be considered “unstimulated”. Unpaired *t* test and 1-way ANOVA was used for comparing between stimulated and unstimulated peptide/samples. *p < 0.05, **p < 0.01, ***p < 0.001

Our study is the first one to examine HLA class I and class II-restricted peptidomes in human PDAC. Previously, similar studies in few other malignant diseases were successfully conducted and reported [6, 10]. This study is also one of the few using MS to identify HLA class II epitopes [11]. Therefore, our study has opened a new direction for the investigation of T cell epitopes and for the development of T cell epitope-based immunotherapy such as vaccine and TCR-T cell therapy in immune “desert” tumors, specifically PDAC [12].

## Supplementary Information

**Additional file 1**: Methods, Supplemental Figures S1–12, and Supplemental Tables S1, S3–5.

**Additional file 2**: Supplemental Table S2, PDAC peptidome.

## Abbreviations

PDAC: Pancreatic ductal adenocarcinoma
MS: Mass spectrometry
TMB: Tumor mutation burdens
HLA-I: HLA class I
HLA-II: HLA class II
FDR: False discovery rate
MFI: Mean fluorescent intensity
SFU: Spot forming unit
