# Supplementary material for: Direct identification of HLA class I and class II-restricted T cell epitopes in pancreatic cancer tissues by mass spectrometry
Source: J Hematol Oncol. 2022 Oct 25;15:154. doi: 10.1186/s13045-022-01373-6 (PMC9597957; doi:10.1186/s13045-022-01373-6)
Supplement: Supplementary file 1 — Additional file 1: Methods, Supplemental Figures S1–12, and Supplemental Tables S1, S3–5. [file 13045_2022_1373_MOESM1_ESM.docx]

**Additional file 1**

**Direct identification of HLA class I and class II-restricted T-cell epitopes in pancreatic cancer tissues by mass spectrometry**

**Authors:** Kenji Fujiwara^1,2,3#^, Yingkuan Shao^1,2,3¶^**^†^**, Nan Niu^1,2,3^, Tengyi Zhang^1,2,3^, Brian Herbst^1,2,3,4^, Mackenzie Henderson^1,2,3^, Stephen Muth^1,2,3^, Pingbo Zhang^6*^, and Lei Zheng^1,2,3,4,5*^

**Affiliations**

^1^ Department of Oncology; Johns Hopkins University School of Medicine; Baltimore, MD 21287; USA.

^2^ The Sidney Kimmel Cancer Center; Johns Hopkins University School of Medicine; Baltimore, MD 21287; USA.

^3^ The Pancreatic Cancer Precision Medicine Center of Excellence Program; Johns Hopkins University School of Medicine; Baltimore, MD 21287; USA.

^4^ The Cellular and Molecular Medicine Graduate Program; Johns Hopkins University School of Medicine; Baltimore, MD 21287; USA.

^5^ Department of Surgery; Johns Hopkins University School of Medicine; Baltimore, MD 21287; USA.

^6^ Department of Ophthalmology; Johns Hopkins University School of Medicine; Baltimore, MD 21287; USA.

***Correspondence:** lzheng6@jhmi.edu, pzhang7@jhmi.edu

**†** Kenji Fujiwara and Yingkuan Shao have contributed equally to this work and should be considered as first authors.

^#^Present address: Department of Surgery, Kimura Hospital, Fukuoka, Japan

^¶^ Present address: The Second Affiliated Hospital, Zhejiang University School of Medicine, Hangzhou, China.

**Methods:**

**Human cancer cell lines and primary human tissues**

Panc 10.05 cells, W6/32 cells, and IVA12 cells, T2 cells were purchased from American Type Culture Collection (ATCC, Manassas, VA) and maintained by following the protocols suggested by ATCC. Panc 06.03 cells had been kept in the laboratory since the establishment of the cell lines[1]. Panc 10.05 and Panc 06.03 are the two cell lines that the irradiated, allogeneic GM-CSF secreting whole cell vaccine (GVAX) derived from[2]. T2-A1 and T2-A3 were genetically modified in our facility from T2 cells, a human B and T lymphoblast hybrid expressing only the HLA-A2 allele, to express the HLA-A1 and HLA-A3 allele[3][4]. Human pancreatic ductal adenocarcinoma (PDAC) resection specimens were obtained from the patients who underwent surgery at the Johns Hopkins Hospital under the Johns Hopkins Medical Institution (JHMI) Institutional Review Board (IRB) approved protocol (IRB00244430) which allows the access of de-identified tumor specimens and peripheral blood mononuclear cells (PBMCs) archived from clinical trial participants who consented for using their specimens for other researches. HLA types, which were the results of PCR tests performed at the clinical laboratory at the Johns Hopkins Medicine, were also part of the archived database associated with the above IRB protocol. Information on biospecimens including PDAC tumor tissues and PBMC was summarized in Table S1.

**Preparation of antibody-conjugated affinity purification columns**

We prepared the antibody-conjugated affinity purification columns by using a modified protocol[5][6][7]. Briefly, we cultured W6/32 cells for pan-HLA-I (A, B, C) antibody and IVA12 cells for pan-HLA-II (DR, DP, DQ) antibody, respectively, and collected the supernatant from the cultures. There is no crossreactivity of this IVA12 antibody towards HLA-I molecules[8]. We applied the supernatants which were diluted in the Pierce Protein A or Protein G binding buffer (Thermo Scientific, Waltham, MA) to the columns packed with the Pierce Protein A Plus Agarose for HLA-I antibodies or Protein G Plus Agarose for HLA-II antibodies (Thermo Scientific), respectively. After washing the columns with the binding buffer and subsequently with the 0.2M sodium borate buffer (pH 9), we cross-linked agarose beads with dimethyl pimelimidate (Thermo Scientific) at the final concentration of 20mM in the sodium borate buffer. After the agarose beads were rotated for 2 hours(h) in the 2.5X beads volumes of 200mM ethanolamine (pH 8), they were washed with the binding buffer and stored in the phosphate-buffered saline at 4°C.

**Purification of HLA bound peptides**

The procedures for purification of HLA bound peptides were modified from those used in the following published studies[5][9]. In brief, the tissues weighted between 100 mg and 1000 mg (Table S1) were immediately frozen in liquid nitrogen after surgical resection and stored at -80°C until the experiment. In preliminary experiments, specimens below 100 mg yielded a suboptimal number of unique peptides while more than 100 mg did not yield higher numbers of peptides (Fig. S12). In contrast, more than 1000 mg yielded peptides that did not peak at 9 mer. The tissue samples used in this study weigh between 100 mg and 680 mg. They were grounded using a mortar and a pestle and incubated in 1-2 ml Pierce IP lysis buffer (Thermo Scientific) containing a Complete Protease Inhibitor Cocktail (Roche, Basel, Switzerland) at 4 °C with constant agitation for 2 h at 4 °C. To lyse cultured cancer cells, 10^8^ cells were pelleted and lysed in 1ml lysis buffer for 1 h. After 30 min centrifugation at 16000g, we incubated the supernatant with unconjugated Protein A beads at 4 °C for 1 h to block the non-specific binding. Then, we incubated the supernatants with the pan-HLA-I antibody-conjugated Protein A beads at the 1/10th lysate volume overnight. After centrifugation, the beads were washed; and then HLA-I bound peptides were eluted as described below. If HLA-II bound peptides were to be purified, after the protein lysate was incubated with the HLA-I antibody conjugated beads to remove the HLA-I bound peptides, the flow-through was used for the isolation of HLA-II bound peptides with the pan-HLA-II antibody-conjugated Protein G beads followed by washing as described above. For peptide elution, The HLA antibody-conjugated beads were washed with Buffer A containing 150 mM NaCl, 20 mM Tris–HCl at a 10X beads volume, Buffer B containing 400 mM NaCl, 20 mM Tris–HCl at a 10X beads volume, Buffer A at a 10X beads volume again, and 20 mM Tris–HCl (pH 8) at a 7X beads volume twice at 4 °C. HLA molecules were eluted at room temperature by 500 μl of 0.1 N acetic acid (pH 3) for 15 minutes. Eluted peptides were loaded to the Sep-Pak C18 3cc/200mg Vac Cartridge (Waters, Milford, MA) which was first activated with 1 mL of methanol and washed with 1 mL 0.1% Trifluoroacetic acid (TFA) three times. The flow-through was repeatedly loaded to the cartridges two more times. The cartridges were washed twice with 300ul of 0.1% TFA. After washing, the peptides were eluted for three times with 400 ul, 300 ul, and 300ul of 80% Acetonitrile in 0.1% TFA, respectively, into a clean 1.5 mL Eppendorf tube. The eluted samples were dried thoroughly at 30°C using vacuum centrifugation and then stored at -80°C.

**LC-MS/MS analysis of HLA peptides**

Above samples were rehydrated in 20 ul of 2% acetonitrile, 0.1% formic acid and placed in an EasyLC autosampler and nanoLC system coupled to an Orbitrap Lumos mass spectrometer (Thermo Fisher). 10 ul of the sample was injected onto a trap column at 5ul/min and then eluted into the mass spectrometer at 300 nl/min over a 90 minute gradient from 2% acetonitrile to 90% acetonitrile in 0.1% formic acid. An in house made nanoLC column (75um ID x 250mm packed with ReproSil-Pur 120 C18-AQ 3um particles (Dr. Maisch, Germany)) was used to separate the peptides. The mass spectrometer was operated at a resolution of 120,000 for MS and 30,000 for MS2. The peptides were fragmented with an isolation window of 1.6 Daltons and collision energy of 30% NCE via higher-energy C-trap dissociation (HCD). As many peptides as possible in a 3-second cycle having a charge of 2-6 were fragmented before doing the next MS precursor scan and precursors which had been previously fragmented were dynamically excluded for 15 seconds. The AGC target for MS was set to 4e5 ions with a maximum injection time of 50 milliseconds and MS2 was set to 1e5 ions and 100 milliseconds maximum. The precursor masses were subjected to calibration on the fly using the Easy-IC fluoranthene lock mass system.

**MS data analysis of HLA peptides**

We used Andromeda of the MaxQuant computational platform, a peptide search engine integrated into the MaxQuant environment (Max Planck Institute of Biochemistry, Munich, Germany)[10][11] to search the peak lists against the UniProt databases (Human 93,609 entries, Feb 2018). We used the settings as suggested in the literature[5][6]. Briefly, the second peptide identification option in Andromeda was enabled. Enzyme specificity was set as unspecific. A false discovery rate of 0.01 was minimally required. The initially allowed mass deviation of the precursor ion was set to 6 p.p.m. The maximum fragment mass deviation was set to 20 p.p.m. We chose 10 out of the 12 processed PDAC samples after excluding Pan03 and Pan08, which may have non-specific HLA-binding peptides as the lengths of the peptides from these samples did not peak unimodally (Fig. S1).We filtered out the peptide sequences that were considered to be reverse sequences or contaminants by MaxQuant. We used NetMHC-4.0 and NetMHCIIpan-4.0 (Department of Health Technology, Lyngby, Denmark) to predict binding affinities of peptides.

**FluoroSpot assay**

The peptides were synthesized and purified to > 95% purity by Peptide 2.0 (Chantilly, VA) according to the sequences identified by MaxQuant. Peptides were stocked in 100% DMSO and diluted in the cell culture medium to yield a final peptide concentration at 10 ng/ml. Archived, cryopreserved PBMCs were recovered and immediately subjected to the FluoroSpot assay. 2x10^5^ PBMCs per well were plated into a 96-well FluoroSpot assay plate from the Human IFN-γ /Granzyme B FluoroSpotPLUS kit or the Human IFN-γ/IL-2/TNF-α FluoroSpotPLUS kit (Mabtech, Cincinnati, OH). Peptides of interest at a concentration of 2 μg/ml were incubated with PBMCs according to the manufacturer’s instruction. Positive controls were PBMCs stimulated with anti-CD3/anti-CD28 antibodies (Mabtech, Cincinnati, OH) or the CEF peptides (Immunospot, Cleveland, OH). Negative controls were PBMC without stimulation. Cytokines produced by PBMCs following peptide stimulation were captured by their specific antibodies conjugated by different fluorescences. Plates were read by an AID iSpot Spectrum reader (Autoimmun Diagnostika GmbH) at the Johns Hopkins University Immune Monitoring Core. The results were processed by the software provided by the manufacturer. Spots that expressed IFN-γ or Granzyme B were counted separately. Spots expressing one, two, or all three cytokines among IFN-γ, IL-2, and TNF-α were counted, respectively.

**Peptide/MHC Binding Assay**

The CEF peptides were used as positive controls (Bio-Synthesis, Lewisville, TX) including CEF1 (GILGFVFTL) and CEF20 (NLVPMVATV) for HLA-A2, CEF24 (VSDGGPNLY) and CEF25 (CTELKLSDY) for HLA-A1, and CEF4 (RVLSFIKGTK) and CEF26 (ILRGSVAHK) for HLA-A3. T2 cells are mono-allelic with HLA-A2. T2-A1, T2-A3, and T2-A11 cells are bi-allelic as they intrinsically express a low amount of HLA-A2 in addition to HLA-A1, A3, and A11, respectively. T2 cells expressing the HLA molecule of interest were resuspended in serum-free AimV medium (Fisher Scientific, Waltham, MA) to a concentration of 10^6^ cells/ml and pulsed with β-2 microglobulin (final concentration at 3ug/ml, Sigma-Aldrich, St. Louis, MO) and peptide (final concentration at 50ug/ml) at room temperature overnight[12]. Cell surface MHC molecules stabilized by the peptide binding were quantified by a CytoFLEX flow cytometer (Beckman Coulter, Brea, CA) with anti-HLA-A2 (Catalog number: 0397HA), A1 (Catalog number: 0289HA), or A3 (Catalog number: 0378HA) mouse monoclonal antibodies (One Lambda, West Hills, CA) as primary antibodies, respectively, and a rabbit anti-mouse FITC-conjugated IgG secondary antibody (Dako, Santa Clara, CA). Dead cells were excluded by being stained with the Live Dead Aqua Dead Cell Kit (Invitrogen). Flow cytometry results were analyzed using the CytExpert software (Beckman Coulter) and were presented as an increase in mean fluorescence intensity (MFI) of cells that were bound with the tested peptide compared to cells without peptide.

**Comparison of the peptide sequences with DNA sequencing**

The Novor software (Rapid Novor Inc, Kitchener, Canada) was used for de novo peptide sequencing, database searching, characterizing unspecific PTMs, and detecting peptide variant sequences according to the user’s manual[13]. After the sequences of peptides were obtained by MS, they were uploaded to the MS-Homology portal (University of California, San Francisco, https://prospector.ucsf.edu/prospector/cgi-bin/msform.cgi?form=mshomology). The number of amino acid differences allowed was set to be one. The protein identities of the resulted peptide sequences were obtained. The Mutalyzer software (Leiden University Medical Center, Leiden, Netherlands, https://mutalyzer.nl/) was used to identify the amino acid changes by comparing the resulted peptide sequences to the wide-type protein sequences. Finally, the peptide variant sequences were compared with the translated protein sequences from the mutated nucleotide sequences according to WES[14].

**Statistical analyses**

All statistical analyses and most of graphs were performed using GraphPad Prism software (GraphPad Software). Venn’s diagrams were drawn with VENNY2.1 (CNB-CSIC, Madrid, Spain). The mean fluorescent intensities or mean values of spot forming units (SFU) in the FluoroSpot assay were compared by Welch’s t-test for two-group comparisons and by one-way ANOVA for multiple group comparisons. If the SFU of a peptide in a sample is less than that of the negative control peptide, it is set as zero; and such a result would be considered “unstimulated”. Because SFUs of negative control peptides vary significantly among different samples, the comparison was made between stimulated and unstimulated peptide/samples. A p-value of less than 0.05 was considered statistically significant.

**Figures and Tables**

**
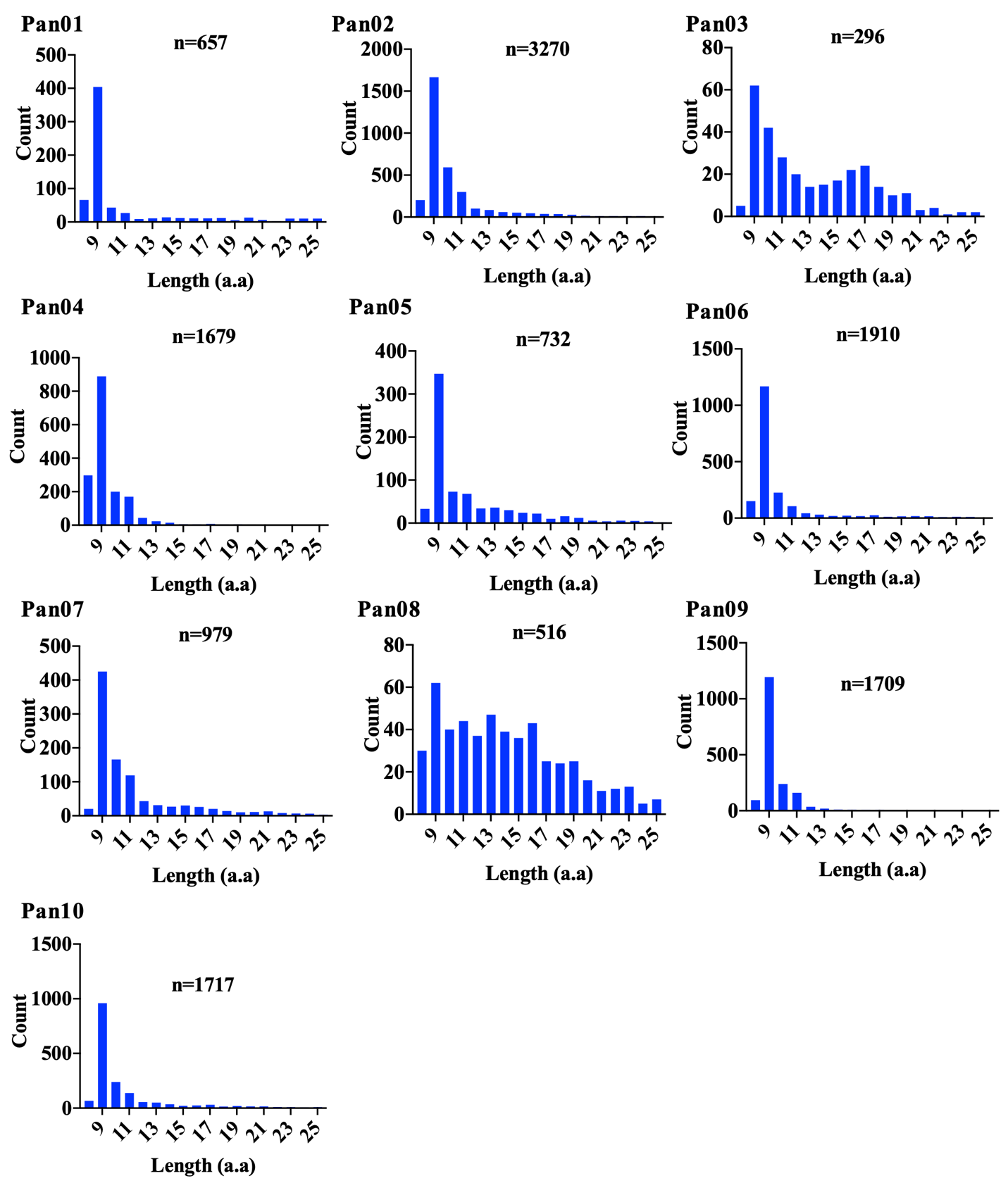
**

**Fig. S1. Additional histograms of the numbers of different lengths of peptides affinity purified by anti-HLA Class I antibody from human PDAC tissues.** We isolated the HLA-I bound peptides from 12 surgically resected human PDAC tissue samples by using the same pan-HLA-I affinity purification column. The peptide-length distribution histograms peaked at 9-mer in all 12 PDAC specimens (Fig. 1B and this supplemental figure). As shown in Fig. 1C, the numbers of eluted peptides from different PDAC specimens varied between 296 and 3270 (1331 on average). These peptides correspond to 123 to 2041 proteins (782 on average), respectively.

**
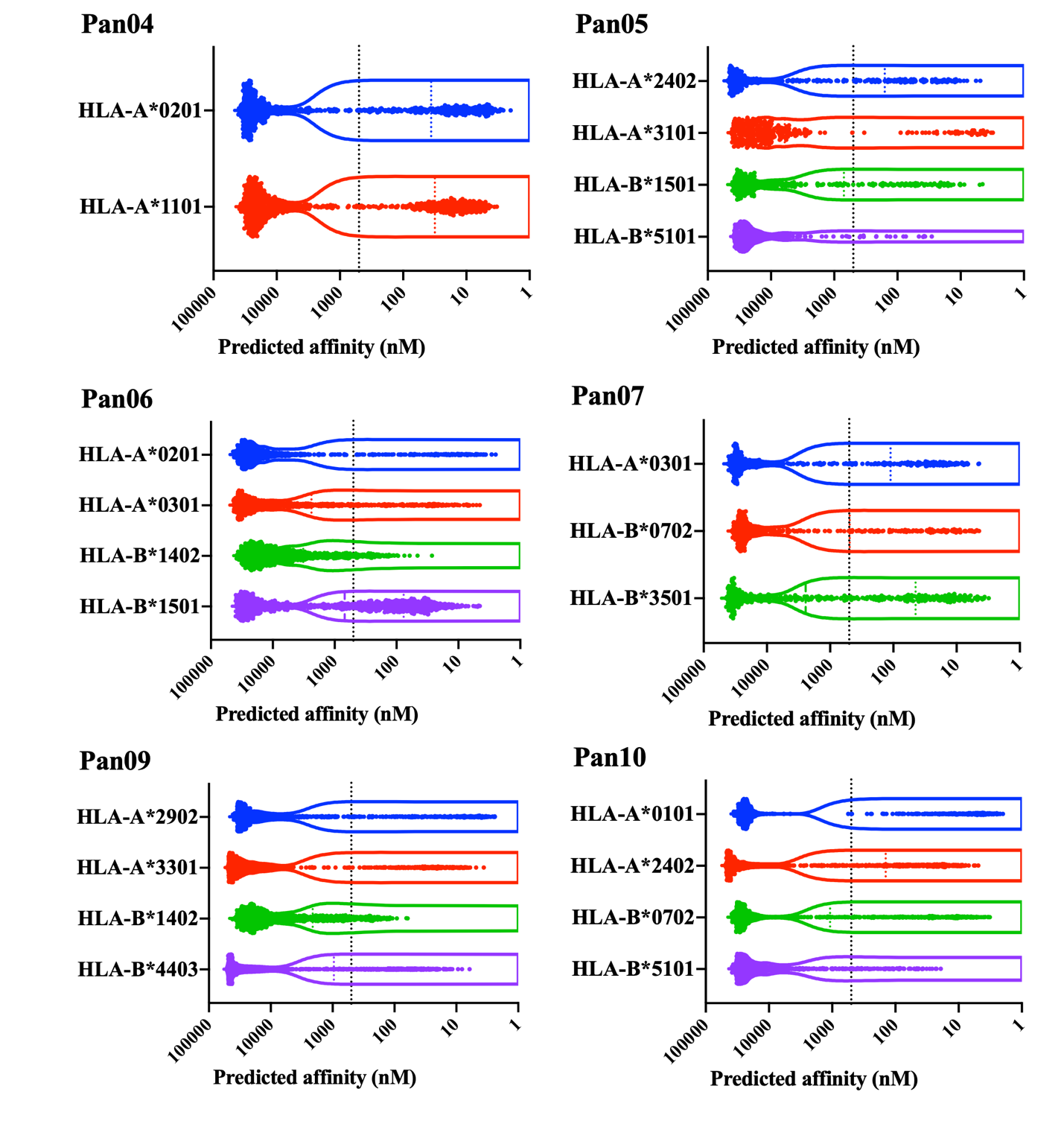
**

**Fig. S2. Predicted H LA Class I binding affinity of eluted peptides from six additional PDAC tissues using the NetMHC4.0 algorithm.** The black dot lines represent the 500 nM threshold of high binding affinity. We examined whether eluted peptides could be predicted to have a high affinity binding to HLA molecules[5]. We filtered the eluted peptides from 8 PDAC specimens to include only 9-mer peptides and predicted their binding affinity to their corresponding HLA-I types by using NetMHC-4.0 (Fig. 1D and this figure)[15]. HLA typing information was available with these 8 out of the 12 processed specimens. The cutoff for the low predicted binding affinity was set as 500 nM (indicated by block dot lines). Note that 339 eluted peptides and 219 eluted peptides from the Pan12 PDAC specimen (81.7% and 52.8% of the total of 415 9-mer peptides, respectively) showed a low predicted binding affinity to the patient’s class I HLA types, HLA-A*2902 and HLA-A*3301, respectively. Similarly, 343 eluted peptides and 319 eluted peptides from the Pan11 PDAC specimen (73.3% and 68.2% of the total of 468 9-mer peptides, respectively) showed a low predicted binding affinity to HLA-A*0101 and HLA-A*2902, respectively.

**
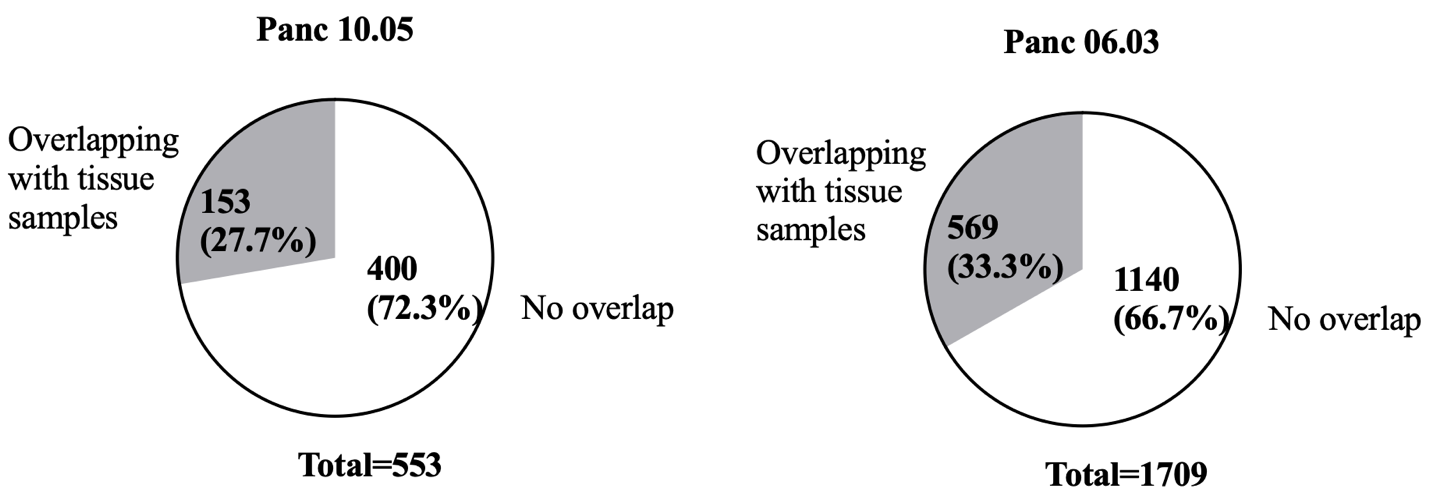
Fig. S3. Numbers of HLA class I peptides from two PDAC cell lines and percentages of overlapping with the whole peptide pool of PDAC tissues.** Note that 153 peptides (27.7%) from Panc 10.05 cells and 569 peptides (33.3%) from Panc 06.03 cells were also found in the peptides eluted from the 10 PDAC tissues.

**
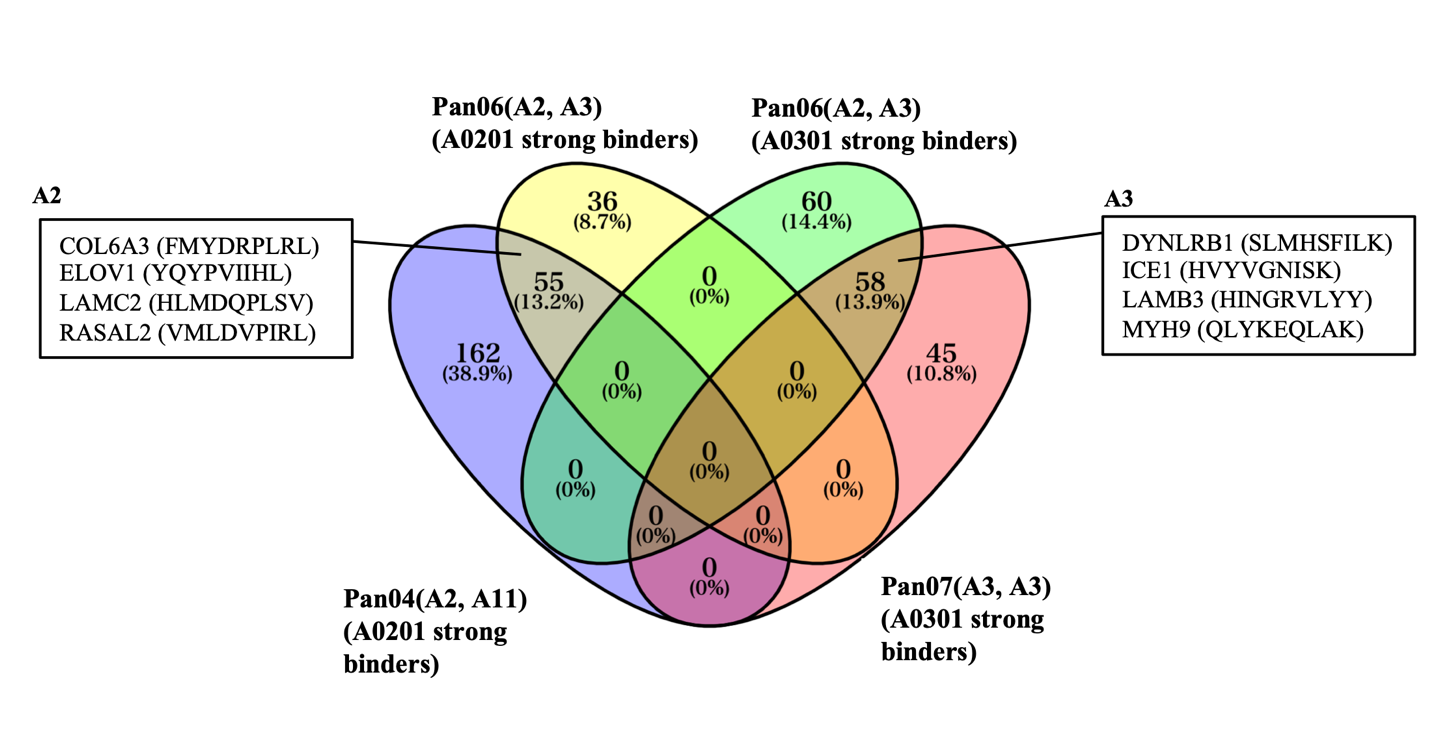
Fig. S4. Numbers of HLA class I peptides defined as strong binders for HLA-A0201and HLA-A0301 of Pan04, Pan06, and Pan07 patients and numbers of overlapping peptides among them.** Whether eluted epitopes were shared among different PDACs were examined in 10 out of the 12 processed PDAC samples after excluding Pan03 and Pan08, which may have non-specific HLA-binding peptides as the lengths of the peptides from these samples did not peak unimodally (Fig. S1).

**
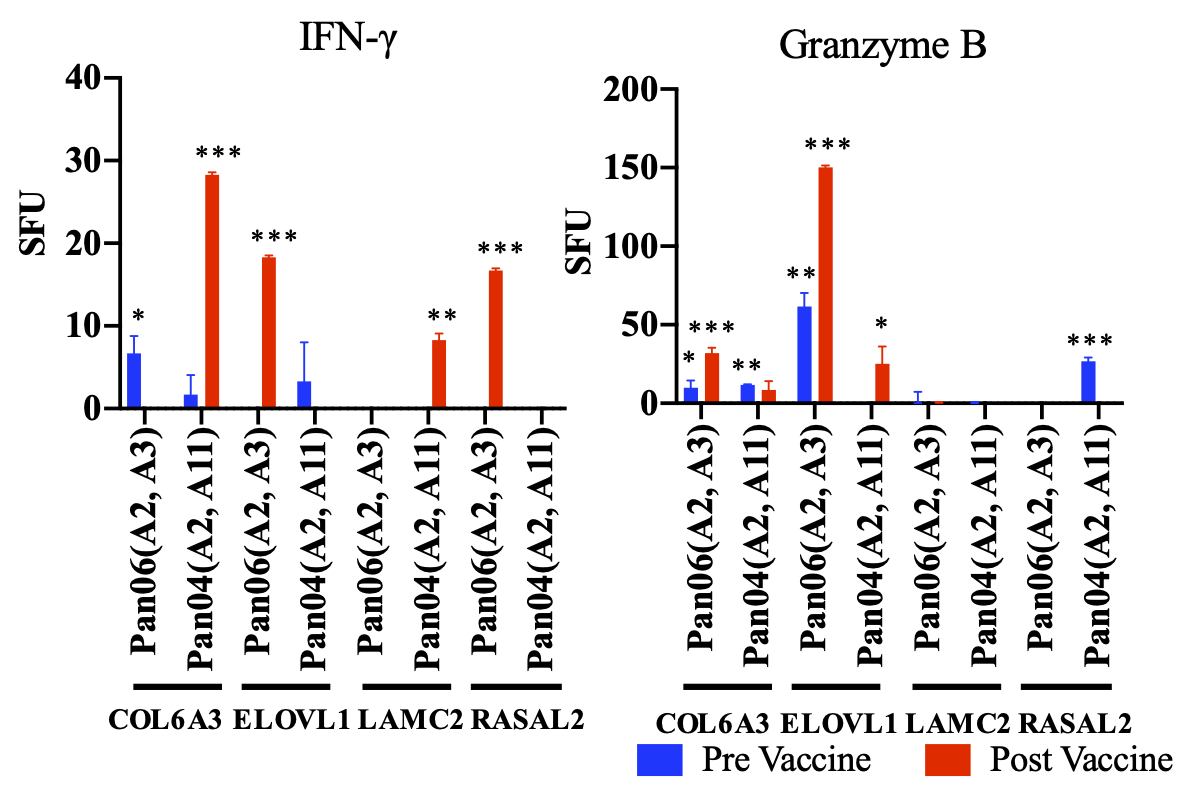
Fig. S5. Validation of selected HLA class I epitopes identified by mass spectrometry in their ability of stimulating T cell responses.** Ability of the synthetic 9-mer peptides in stimulating the IFN-γ and granzyme B expression from T cells in PBMCs from HLA-A2 patients including Pan06 (HLA-A*0201, A*0301) and Pan04 (HLA-A*0201, A*1101) in a FluoroSpot assay was shown in the histograms. Pre vaccine: PBMCs collected before the patients received the GM-CSF-secreting, allogeneic pancreatic tumor whole cell vaccine (PDAC GVAX vaccine) therapy. Post vaccine: PBMCs collected after the patients received the PDAC GVAX vaccine therapy. For this study, we do not intend to use PBMCs from the patients who received the GVAX vaccine. However, many patients at JHMI received the GVAX vaccine. The PBMC samples were always archived before and after the PDAC patients received the first treatment of GVAX through the past clinical trials and available for other researches under the JHMI IRB general banking protocol. More specifically, the PBMC samples used were obtained from the HLA-A2 (Pan06 and Pan04) and A3 patients (Pan06) whose tumors were used for identifying these peptides[16]. Similarly, the tumor specimens archived under the JHMI IRB-approved general banking protocol and used in this study happened to be obtained from patients who underwent the surgical resection following the treatment of GVAX. However, we do not anticipate that the treatment of GVAX, which is made of irradiated, allogenic whole tumor cells[17], would have an impact on the identification of epitopes; however, archived biospecimen repositories of clinical trials would provide PBMCs to compare peripheral T cell response at different time points. We do anticipate that the treatment of GVAX, which expresses many epitopes that were identified in the PDAC tissues (Fig. S3), would enhance the peripheral T cell response to these epitopes. The results showed that T cell response as demonstrated by the expression of either IFN-γ or granzyme B or both was significantly stimulated by each of the eight selected peptides in the PBMC from at least one of two patients whose tumors were used for identifying these peptides. Note that T cell response was also stimulated by the peptides in the PBMC samples from other HLA type-matched patients. As anticipated, T cell response was observed in the PBMC collected before receiving the GVAX vaccine. However, T cell response was more likely observed in the PBMC collected after receiving the GVAX vaccine, suggesting this whole cell vaccine expresses at least some of the shared antigens and thus was able to induce the proliferation of T cells specific for those antigens. Nevertheless, T cell response in some of the peptides was decreased in the PBMC collected after receiving the GVAX vaccine, suggesting the GVAX vaccine treatment did not adequately present every one of these 8 epitopes. Spot forming unit (SFU) is the number of spots per 10^6^ PBMCs. Unpaired t test and 1-way ANOVA was used for comparing between stimulated and unstimulated peptide/samples. *p < 0.05, **p < 0.01, ***p < 0.001.

**
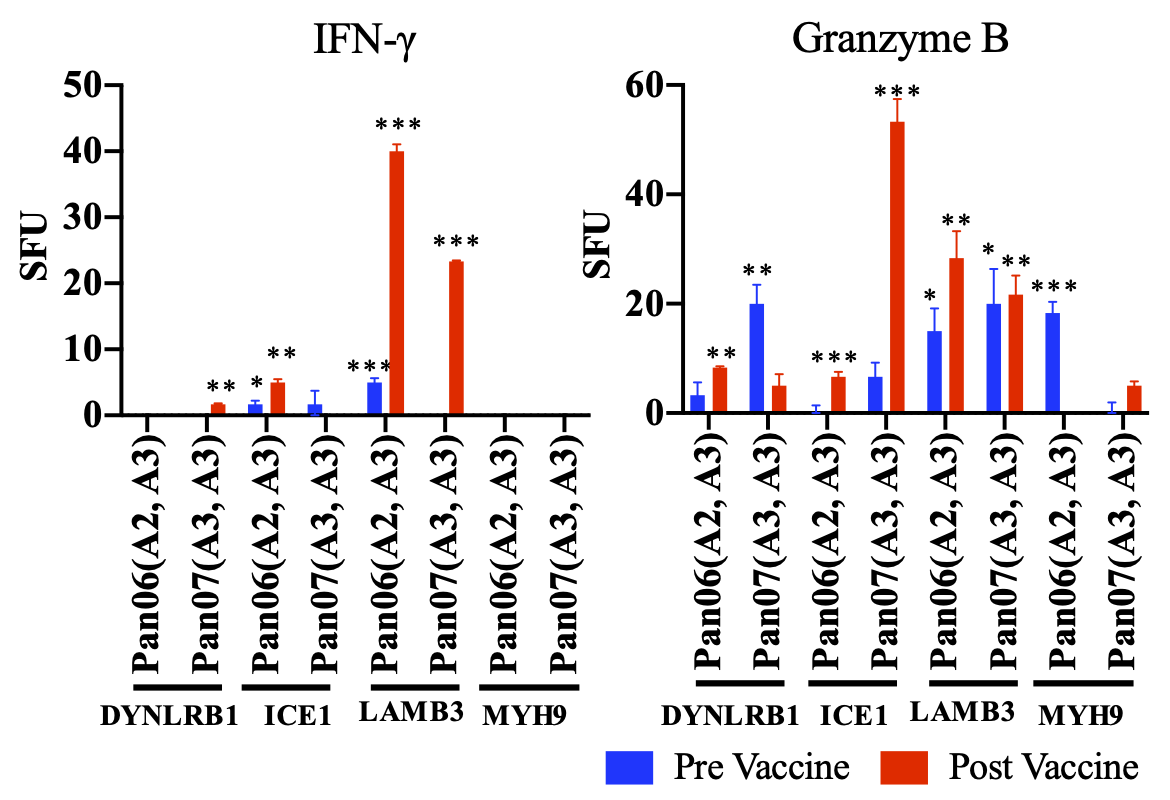
Fig. S6. Ability of the synthetic 9-mer peptides in stimulating the IFN-γ and granzyme B expression from T cells in PBMCs from HLA-A3 patients in a FluoroSpot assay was shown in the histograms.** Pre vaccine: PBMCs collected before the patients including Pan06 (HLA-A*0201, A*0301) and Pan07 (HLA-A*0301) received the PDAC GVAX vaccine therapy. Post vaccine: PBMCs collected after the patients (Pan06 and Pan07) received the PDAC GVAX vaccine therapy. Spot forming unit (SFU) is the number of spots per 10^6^ PBMCs. Unpaired t test and 1-way ANOVA was used for comparing between stimulated and unstimulated peptide/samples. *p < 0.05, **p < 0.01, ***p < 0.001.

**
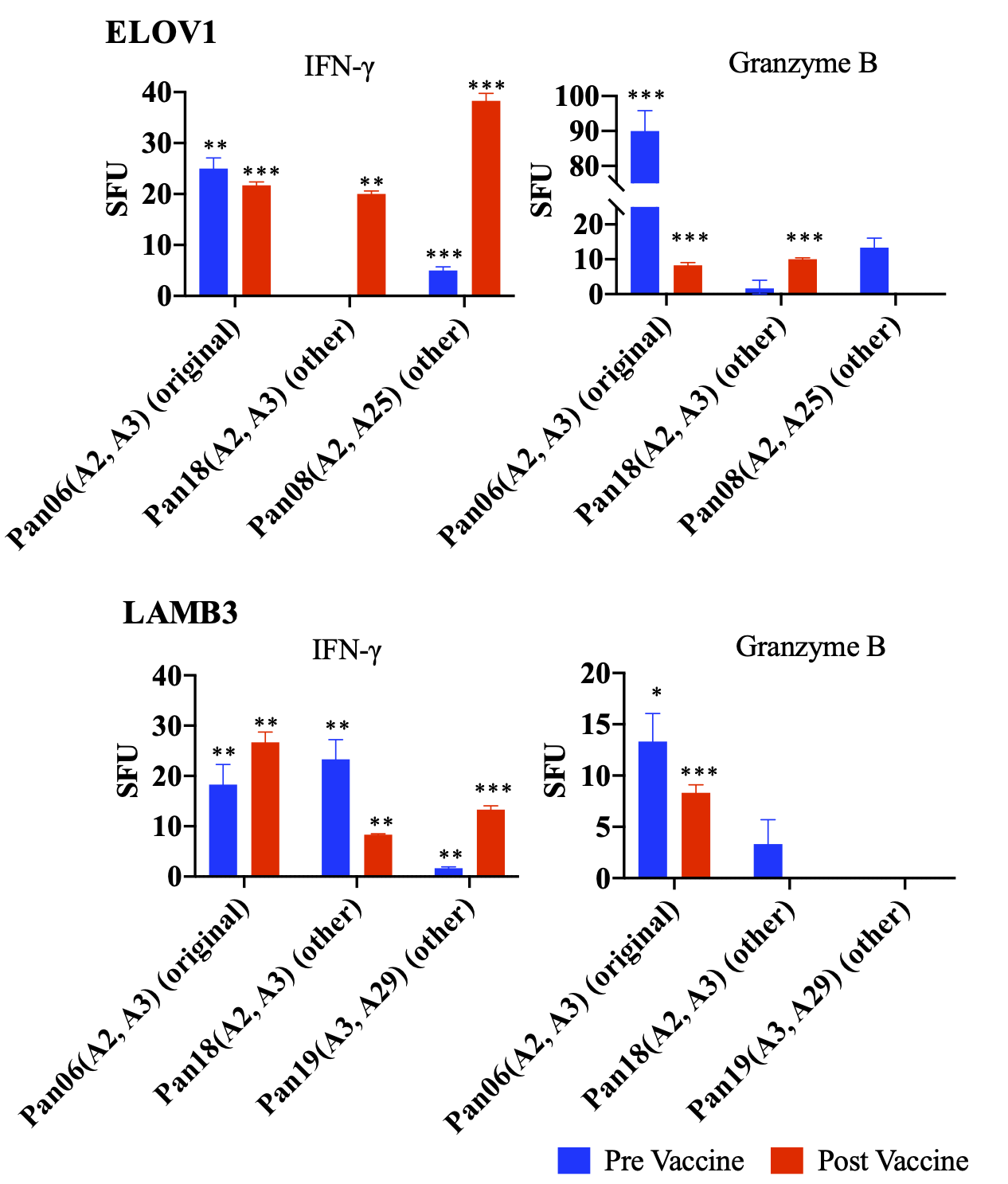
Fig. S7. Ability of two representative peptides ELOVL1 and LAMB3 in stimulating the IFN-γ and granzyme B expression from T cells in a FluoroSpot assay.** PBMCs from the patient (designated “original”: Pan06 (HLA-A*0201, A*0301)) where the peptides were eluted from and those from other patients (designated “other”: Pan18 (HLA-A*0201, A*0301); Pan08 (HLA-A*0201, A*2501); Pan19 (HLA-A*0301, A*2902)) were tested. Pre vaccine: PBMCs collected before the patients received the PDAC GVAX vaccine therapy. Post vaccine: PBMCs collected after the patients received the PDAC GVAX vaccine therapy. Spot forming unit (SFU) is the number of spots per 10^6^ PBMCs. Unpaired t test and 1-way ANOVA was used for comparing between stimulated and unstimulated peptide/samples. *p < 0.05, **p < 0.01, ***p < 0.001.

**
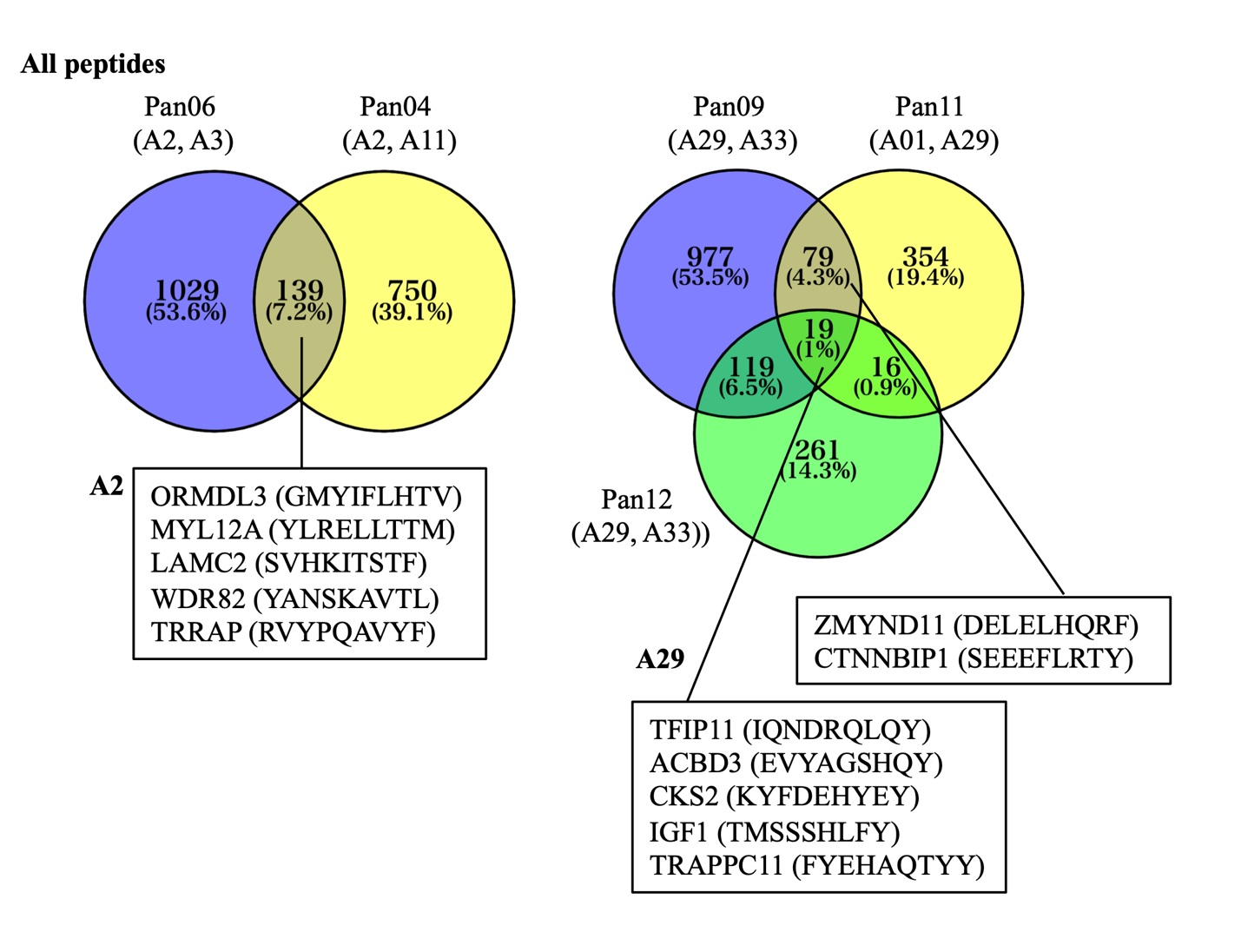
Fig. S8. Numbers of HLA class I peptides of Pan06 and Pan04 patients, and overlapped peptides between patients were indicated (left). Peptide numbers of Pan09, Pan11, and Pan12 patients, and overlapping peptide numbers among patients (right).** Note that we compared both HLA-I and HLA-II peptide sequences with the DNA WES results available from 4 PDACs and did not find any peptide sequence matched to the nucleotide sequence variants including single nucleotide polymorphism (SNP). It is possible that HLA-bound peptide identification may have missed the mutations-associated neoepitopes. However, such a result is consistent with the known rareness of the genomic mutation-associated neoepitopes in PDAC[18].

**
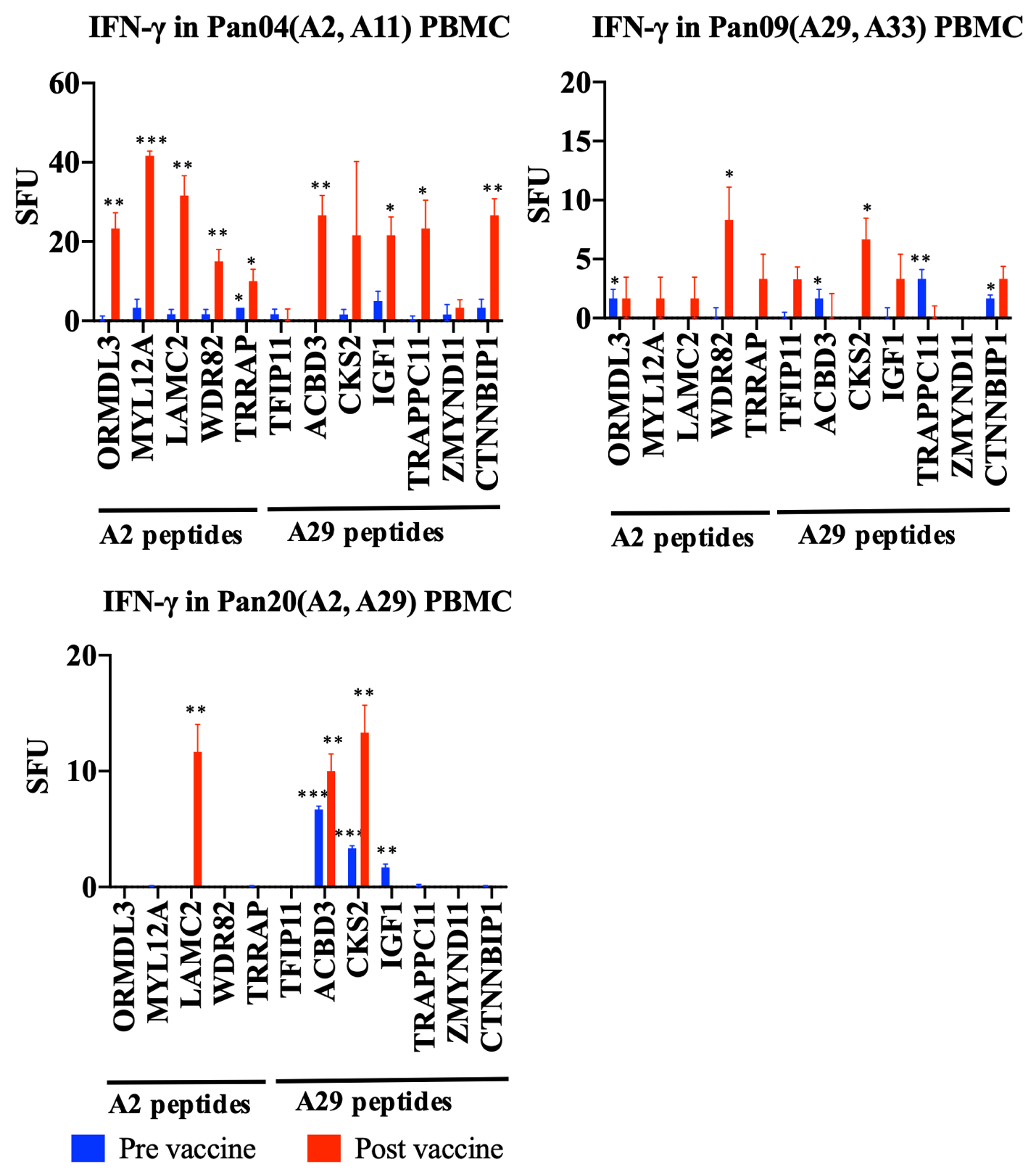
Fig. S9. Validation of selected HLA class I epitopes identified by mass spectrometry in their ability of binding to unmatched HLA class I molecules and stimulating T cell responses in unmatched PBMC.**Ability of selected 9-mer peptides in stimulating the IFN-γ expression from T cells in PBMCs from patients including Pan04 (HLA-A*0201, A*1101), Pan09 (HLA-A*2902, A*3301), and Pan20 (HLA-A*0201, A*2902) with HLA class I types indicated, respectively, in a FluoroSpot assay, was shown in the histograms. The PBMC samples were archived before and after the PDAC patients received the first treatment of GVAX through the past clinical trials and available for other researches under the JHMI IRB general banking protocol.  MFI: mean fluorescent intensity. Pre vaccine: PBMCs collected before the patients received the PDAC GVAX vaccine therapy. Post vaccine: PBMCs collected after the patients received the PDAC GVAX vaccine therapy. Spot forming unit (SFU) is the number of spots per 10^6^ PBMCs. Shown is SFU of each peptide after subtracting that of a negative control peptide; and error bars represent the percentages of deviation. If the SFU of a peptide in a sample is less than that of the negative control peptide, it is set as zero; and such a result would be considered “unstimulated”.  Unpaired t test and 1-way ANOVA was used for comparing between stimulated and unstimulated peptide/samples. *p < 0.05, **p < 0.01, ***p < 0.001.

**
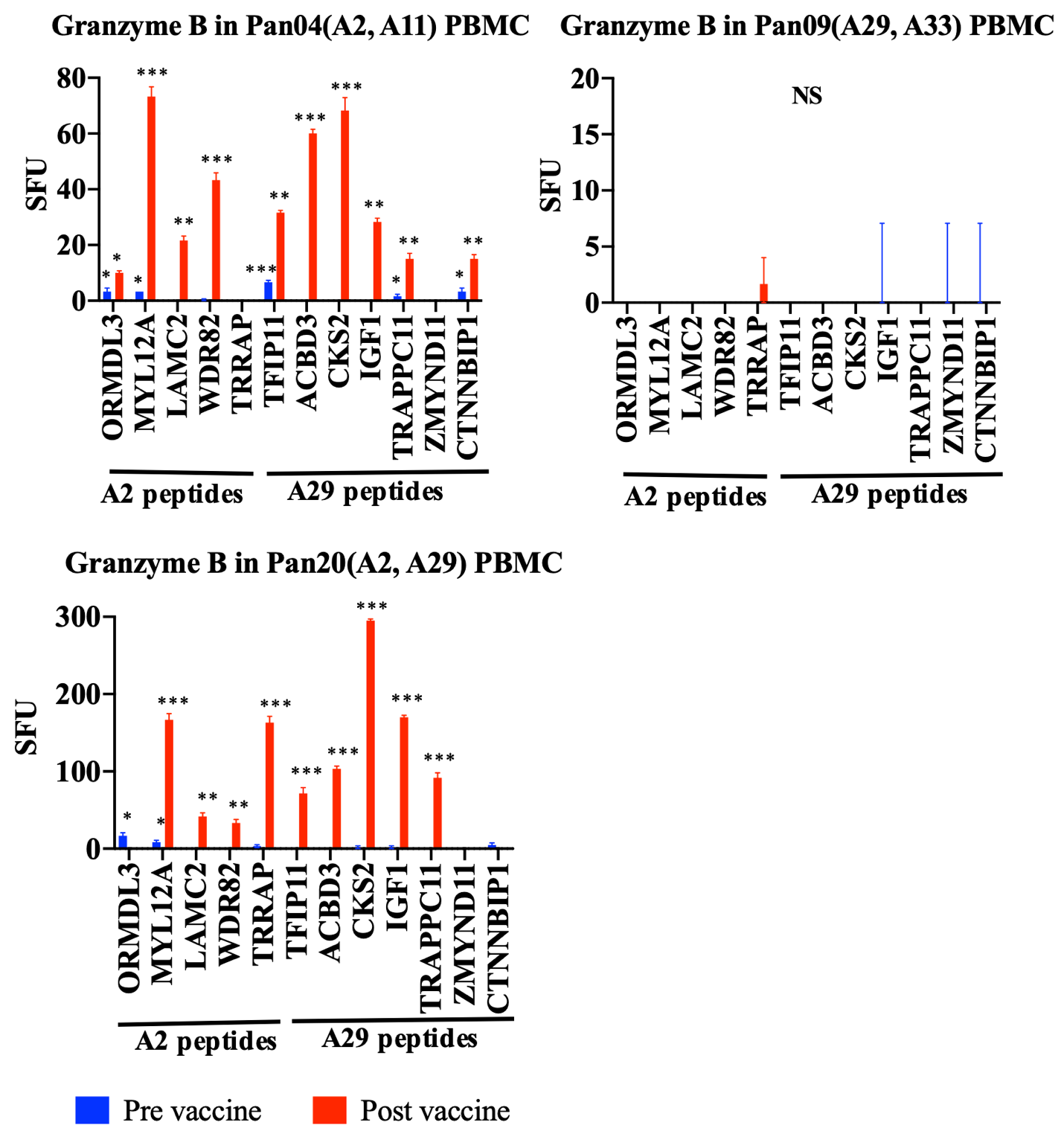
Fig. S10. Ability of selected 9-mer peptides in stimulating the granzyme B expression from T cells in PBMCs from patients with HLA class I types indicated, respectively, in a FluoroSpot assay, was shown.**MFI: mean fluorescent intensity. Pre vaccine: PBMCs collected before the patients received the PDAC GVAX vaccine therapy. Post vaccine: PBMCs collected after the patients received the PDAC GVAX vaccine therapy. Note that the HLA-A29 peptides were able to stimulate T cells from both Pan04 (HLA-A*0201, A*1101), a non-HLA-A29 patient, and Pan20 (HLA-A*0201, A*2902), an HLA-A29 patient; and the HLA-A2 peptides were able to stimulate T cells from Pan09 (HLA-A*2902, A*3301), a non-HLA-A2 patient. Thus, our results suggested that above identified HLA-A2 and -A29 epitopes are able to bind unmatched HLA molecules and stimulate the T cell response in HLA-unmatched PBMC samples.  Spot forming unit (SFU) is the number of spots per 10^6^ PBMCs. Shown is SFU of each peptide after subtracting that of a negative control peptide; and error bars represent the percentages of deviation. If the SFU of a peptide in a sample is less than that of the negative control peptide, it is set as zero; and such a result would be considered “unstimulated”.  Unpaired t test and 1-way ANOVA was used for comparing between stimulated and unstimulated peptide/samples. *p < 0.05, **p < 0.01, ***p < 0.001.

**
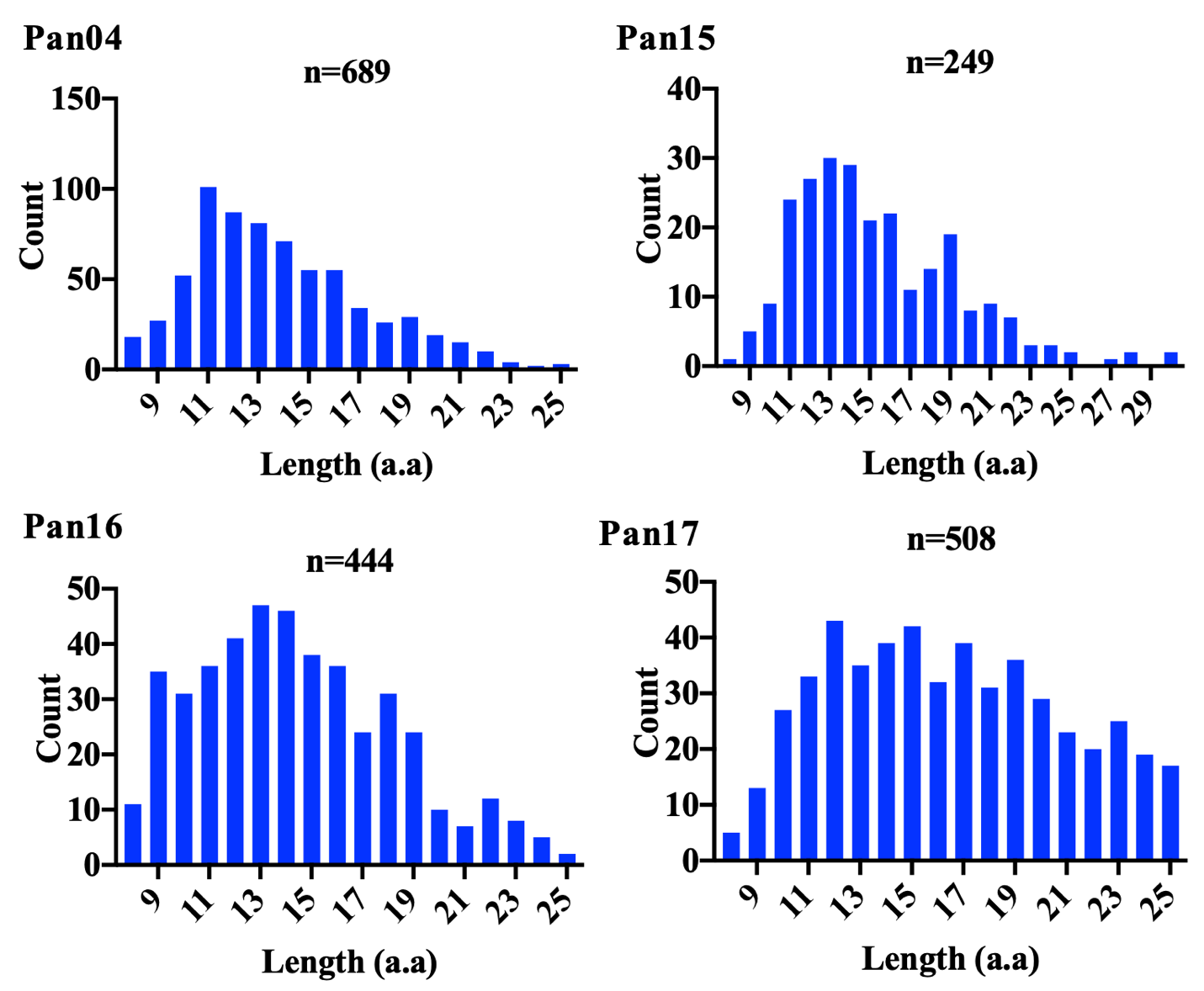
Fig. S11. The histograms of the numbers of different lengths of peptides affinity purified by anti-HLA Class II antibody from human PDAC tissues.**

**
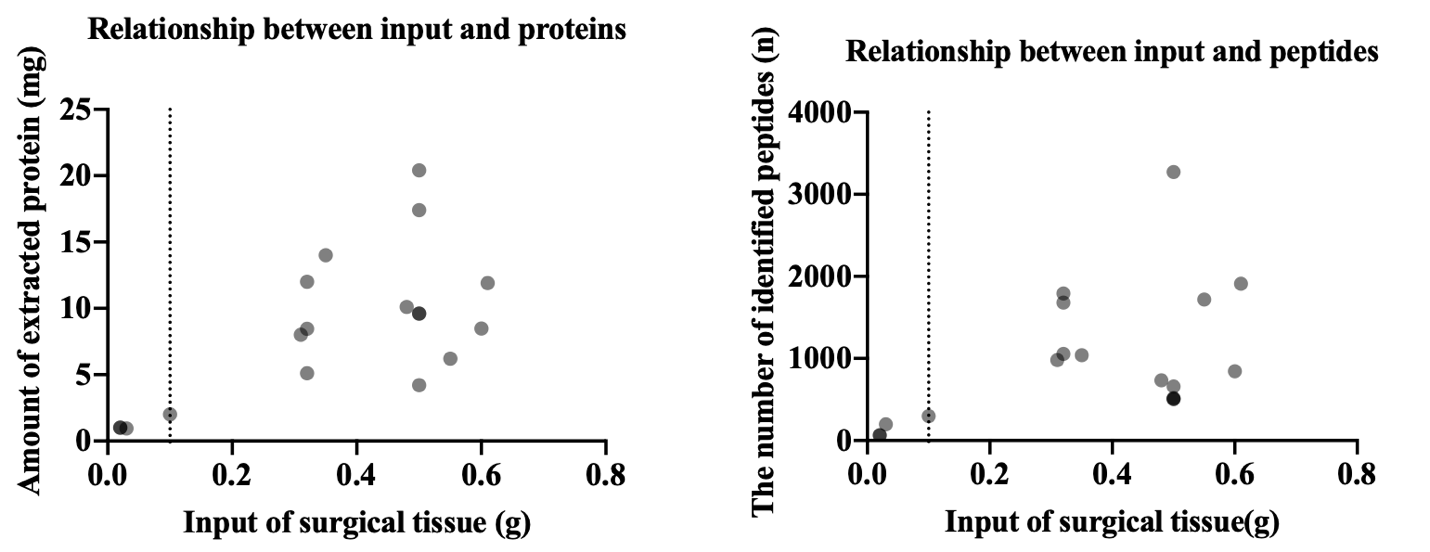
Fig. S12. The relationship between the input of surgical tissue and the amount of extracted protein (left) and between the input of surgical tissue and the number of identified peptides (left).** The black dot lines represent 100mg input of surgical tissue.

**Table S1. Summary of Biospecimen Information.**

| Patient ID | Biospecimen used | Weight of tumor tissue (mg) | Numbers of identified peptides | | HLA types | | | | | | | |
| --- | --- | --- | --- | --- | --- | --- | --- | --- | --- | --- | --- | --- |
|  |  |  | HLA Class I | HLA Class II | HLA-A | HLA-A | HLA-B | HLA-B | HLA-C | HLA-C | HLA-DRB1 | HLA-DRB1 |
| Pan01 | Tumor tissue | 500 | 657 | NA | NA | NA | NA | NA | NA | NA | NA | NA |
| Pan02 | Tumor tissue | 500 | 3271 | NA | NA | NA | NA | NA | NA | NA | NA | NA |
| Pan03 | Tumor tissue | 100 | 296 | NA | NA | NA | NA | NA | NA | NA | NA | NA |
| Pan04 | Tumor tissue, PBMC | 320 | 1679 | 689 | HLA-A*0201 | HLA-A*1101 | HLA-B*5201 | HLA-B*5201 | HLA-C*1202 | HLA-C*1202 | HLA-DRB1*0405 | HLA-DRB1*1103 |
| Pan05 | Tumor tissue | 480 | 732 | NA | HLA-A*2402 | HLA-A*3101 | HLA-B*1501 | HLA-B*5101 | HLA-C*0303 | HLA-C*1402 | NA | NA |
| Pan06 | Tumor tissue, PBMC | 610 | 1909 | NA | HLA-A*0201 | HLA-A*0301 | HLA-B*1402 | HLA-B*1501 | HLA-C*0303 | HLA-C*0802 | NA | NA |
| Pan07 | Tumor tissue, PBMC | 310 | 979 | NA | HLA-A*0301 | HLA-A*0301 | HLA-B*0702 | HLA-B*3501 | HLA-C*0401 | HLA-C*0702 | NA | NA |
| Pan08 | Tumor tissue, PBMC | 500 | 516 | NA | HLA-A*0201 | HLA-A*2501 | HLA-B*3801 | HLA-B*5101 | HLA-C*0701 | HLA-C*1203 | NA | NA |
| Pan09 | Tumor tissue, PBMC | 320 | 1791 | NA | HLA-A*2902 | HLA-A*3301 | HLA-B*1402 | HLA-B*4403 | HLA-C*0802 | HLA-C*1601 | NA | NA |
| Pan10 | Tumor tissue | 550 | 1717 | NA | HLA-A*0101 | HLA-A*2402 | HLA-B*0702 | HLA-B*5101 | HLA-C*0102 | HLA-C*0702 | NA | NA |
| Pan11 | Tumor tissue | 320 | 1054 | NA | HLA-A*0101 | HLA-A*2902 | HLA-B*3502 | HLA-B*4403 | HLA-C*0401 | HLA-C*1601 | NA | NA |
| Pan12 | Tumor tissue | 600 | 843 | NA | HLA-A*2902 | HLA-A*3301 | HLA-B*0702 | HLA-B*6501 | HLA-C*0701 | HLA-C*0802 | NA | NA |
| Pan13 | Tumor tissue | 590 | NA | 665 | HLA-A*1101 | HLA-A*2601 | HLA-B*4901 | HLA-B*5701 | HLA-C*0501 | HLA-C*0601 | HLA-DRB1*0801 | HLA-DRB1*1303 |
| Pan14 | Tumor tissue | 672 | NA | 450 | HLA-A*0201 | HLA-A*2402 | HLA-B*4502 | HLA-B*6201 | HLA-C*0401 | HLA-C*1201 | HLA-DRB1*0103 | HLA-DRB1*0701 |
| Pan15 | Tumor tissue, PBMC | 550 | 109 | 249 | HLA-A*0101 | HLA-A*2301 | HLA-B*0802 | HLA-B*1303 | HLA-C*1001 | HLA-C*1801 | HLA-DRB1*1101 | HLA-DRB1*0801 |
| Pan16 | Tumor tissue, PBMC | 678 | 1236 | 444 | HLA-A*0201 | HLA-A*2402 | HLA-B*3501 | HLA-B*3701 | HLA-C*0401 | HLA-C*0602 | HLA-DRB1*1418 | HLA-DRB1*0304 |
| Pan17 | Tumor tissue | 845 | 184 | 508 | HLA-A*0101 | HLA-A*0201 | HLA-B*0801 | HLA-B*1301 | HLA-C*0601 | HLA-C*0701 | HLA-DRB1*0401 | HLA-DRB1*1703 |
| Pan18 | PBMC | NA | NA | NA | HLA-A*0201 | HLA-A*0301 | HLA-B*4001 | HLA-B*4403 | HLA-C*0304 | HLA-C*1601 | NA | NA |
| Pan19 | PBMC | NA | NA | NA | HLA-A*0301 | HLA-A*2902 | HLA-B*4901 | HLA-B*5501 | HLA-C*0303 | HLA-C*0701 | NA | NA |
| Pan20 | PBMC | NA | NA | NA | HLA-A*0201 | HLA-A*2902 | HLA-B*4402 | HLA-B*4403 | HLA-C*0501 | HLA-C*1601 | NA | NA |

**Table S2. PDAC Peptidome.**

(Uploaded as an Excel file)

**Table S3. Number of PDACs that share the epitopes and number of epitopes that are shared.**

| **Number of PDACs**  **that share the epitopes** | **Number (%) of unique peptides**  **shared (Total number: 6553)** | **Number (%) of unique proteins**  **shared (Total number: 4444)** |
| --- | --- | --- |
| 1 | 5390 (82.3%) | 3099 (69.7%) |
| 2 | 989 (15.1%) | 875 (19.7%) |
| 3 | 148 (2.3%) | 281 (6.3%) |
| 4 | 15 (0.23%) | 114 (2.6%) |
| 5 | 8 (0.12%) | 37 (0.83%) |
| 6 | 2 (0.03%) | 16 (0.36%) |
| 7 | 1 (0.02%) | 11 (0.25%) |

**Table S4. Selected HLA-A2 peptides and A3 peptides.**

| Predicted HLA type* | Sequence | Gene name | HLA types of patients where the epitopes derived | A0201 Rank** | A0301 Rank** | | A2902 Rank** | | A0101 Rank* | |
| --- | --- | --- | --- | --- | --- | --- | --- | --- | --- | --- |
| Epitopes ranked according to the NetMHC predicted binding affinity to HLA-A0201 or –A0301 | | | | | | | |  | |  |
| A2 | FMYDRPLRL | COL6A3 | 1) A0201,0301 2)A0201,1101 | 0.03 | 5 | |  | |  | |
| A2 | YQYPVIIHL | ELOVL1 | 1) A0201,0301 2)A0201,1101 | 0.175 | 10 | |  | |  | |
| A2 | HLMDQPLSV | LAMC2 | 1) A0201,0301 2)A0201,1101 | 0.05 | 5.5 | |  | |  | |
| A2 | VMLDVPIRL | RASAL2 | 1) A0201,0301 2)A0201,1101 | 0.1 | 11 | |  | |  | |
| A3 | SLMHSFILK | DYNLRB1 | 1) A0201,0301 2) A0301  3) A0201,1101 | 8 | 0.01 | |  | |  | |
| A3 | HVYVGNISK | ICE1 | 1) A0201,0301 2) A0301 | 38 | 0.125 | |  | |  | |
| A3 | HINGRVLYY | LAMB3 | 1) A0201,0301 2) A0301 | 26 | 0.175 | |  | |  | |
| A3 | QLYKEQLAK | MYH9 | 1) A0201,0301 2) A0301 | 29 | 0.1 | |  | |  | |
| Epitopes shared by multiple PDACs with different HLA types | | | | | |  | |  | |  |
| A2 | GMYIFLHTV | ORMDL3 | 1) A0201,0301 2)A0201,1101 | 0.1 | 6.5 | | 3.5 | |  | |
| A2 | YLRELLTTM | MYL12A | 1) A0201,0301 2)A0201,1101 | 0.9 | 15 | | 3 | |  | |
| A2 | SVHKITSTF | LAMC2 | 1) A0201,0301 2)A0201,1101 | 30 | 9.5 | | 5.5 | |  | |
| A2 | YANSKAVTL | WDR82 | 1) A0201,0301 2)A0201,1101 | 7.5 | 29 | | 20 | |  | |
| A2 | RVYPQAVYF | TRRAP | 1) A0201,0301 2)A0201,1101 | 16 | 1.5 | | 0.9 | |  | |
| A29 | IQNDRQLQY | TFIP11 | 1) A2902, A3301 2) A2902, A0101 3) A29, A33 | 44 |  | | 0.8 | | 1.8 | |
| A29 | EVYAGSHQY | ACBD3 | 1) A2902, A3301 2) A2902, A0101 3) A29, A33 | 60 |  | | 0.1 | | 2.5 | |
| A29 | KYFDEHYEY | CKS2 | 1) A2902, A3301 2) A2902, A0101 3) A29, A33 | 60 |  | | 0.04 | | 12 | |
| A29 | TMSSSHLFY | IGF1 | 1) A2902, A3301 2) A2902, A0101 3) A29, A33 | 23 |  | | 0.01 | | 0.04 | |
| A29 | FYEHAQTYY | TRAPPC11 | 1) A2902, A3301 2) A2902, A0101 3) A29, A33 | 60 |  | | 0.3 | | 1.2 | |
| A29 | DELELHQRF | ZMYND11 | 1) A2902, A3301 2) A2902, A0101 | 65 |  | | 15 | | 26 | |
| A29 | SEEEFLRTY | CTNNBIP1 | 1) A2902, A3301 2) A2902, A0101 | 41 |  | | 6.5 | | 20 | |

*If the patient had a heterozygosity of HLA class I alleles, the peptides would be classified according to the allele with a higher binding affinity predicted by NetMHC.

**Rank<0.5: strong predicted binding affinity

**Table S5. Six synthesized HLA class II peptides.**

| **Peptide sequence** | **Length of peptides** | **Gene names** | **PDAC expression** | **Normal Tissue expression** | **Predicted binding affinity (nM)**  **(DRB1_0113-restricted)** |
| --- | --- | --- | --- | --- | --- |
| ADSGEGDFLAEGGGVR | 16-mer | FGA | Low | All | 1443.6 |
| NSGALTSGVHTFPAVLQS | 18-mer | IGHG | NA | NA | 914.2 |
| TLPTKETIEQEKRSEIS | 17-mer | TMSB10 | Medium | Blood | 6807.9 |
| TVETRDGQVINETSQHHDDLE | 21-mer | VIM | Medium | Liver | 2331.4 |
| VVAGVANALAHK | 12-mer | HBD | Low | Bone marrow | 356.5 |
| SGPPVSELITKAVAASKER | 19-mer | H1F2 | Medium | Lymphoid tissue | 73.1 |

**References in Additional File 1**

1. Jaffee EM, Schutte M, Gossett J, Morsberger LA, Adler AJ, Thomas M, et al. Development and characterization of a cytokine-secreting pancreatic adenocarcinoma vaccine from primary tumors for use in clinical trials. Cancer J Sci Am. United States; 1998;4:194–203.

2. Le DT, Wang-Gillam A, Picozzi V, Greten TF, Crocenzi T, Springett G, et al. Safety and survival with GVAX pancreas prime and Listeria Monocytogenes-expressing mesothelin (CRS-207) boost vaccines for metastatic pancreatic cancer. J Clin Oncol. 2015;33:1325–33.

3. Salter RD, Howell DN, Cresswell P. Genes regulating HLA class I antigen expression in T-B lymphoblast hybrids. Immunogenetics. United States; 1985;21:235–46.

4. Anderson KS, Alexander J, Wei M, Cresswell P. Intracellular transport of class I MHC molecules in antigen processing mutant cell lines. J Immunol. United States; 1993;151:3407–19.

5. Bassani-Sternberg M, Bräunlein E, Klar R, Engleitner T, Sinitcyn P, Audehm S, et al. Direct identification of clinically relevant neoepitopes presented on native human melanoma tissue by mass spectrometry. Nat Commun. 2016;7:1–16.

6. Bassani-Sternberg M. Mass Spectrometry Based Immunopeptidomics for the Discovery of Cancer Neoantigens. Methods Mol Biol. United States; 2018;1719:209–21.

7. Rozanov D V, Rozanov ND, Chiotti KE, Reddy A, Wilmarth PA, David LL, et al. MHC class I loaded ligands from breast cancer cell lines: A potential HLA-I-typed antigen collection. J Proteomics. 2018;176:13–23.

8. Shaw S, Ziegler A, DeMars R. Specificity of monoclonal antibodies directed against human and murine class II histocompatibility antigens as analyzed by binding to HLA-deletion mutant cell lines. Hum Immunol. United States; 1985;12:191–211.

9. Kalaora S, Barnea E, Merhavi-Shoham E, Qutob N, Teer JK, Shimony N, et al. Use of HLA peptidomics and whole exome sequencing to identify human immunogenic neo-antigens. Oncotarget. 2016;7:5110–7.

10. Cox J, Mann M. MaxQuant enables high peptide identification rates, individualized p.p.b.-range mass accuracies and proteome-wide protein quantification. Nat Biotechnol. United States; 2008;26:1367–72.

11. Cox J, Neuhauser N, Michalski A, Scheltema RA, Olsen J V, Mann M. Andromeda: a peptide search engine integrated into the MaxQuant environment. J Proteome Res. United States; 2011;10:1794–805.

12. Thomas AM, Santarsiero LM, Lutz ER, Armstrong TD, Chen Y-C, Huang L-Q, et al. Mesothelin-specific CD8 + T Cell Responses Provide Evidence of In Vivo Cross-Priming by Antigen-Presenting Cells in Vaccinated Pancreatic Cancer Patients . J Exp Med. 2004;200:297–306.

13. Ma B. Novor: real-time peptide de novo sequencing software. J Am Soc Mass Spectrom. 2015;26:1885–94.

14. Li K, Tandurella JA, Gai J, Zhu Q, Lim SJ, Thomas IIDL, Xia T, Mo G, Mitchell JT, Montagne J, Lyman M, Danilova LV, Zimmerman JW, Kinny-Köster B, Zhang T, Chen L, Blair AB, Heumann T, Parkinson R, DurhamJN, Narang A, Anders RA, Wolfgang CL, Laheru DA, He J, Osipov A, Thompson ED, Wang H, Fertig EJ, Jaffee EM, Zheng L. Multi-omics analyses of changes in the tumor microenvironment of pancreatic adenocarcinoma following neoadjuvant treatment with anti-PD-1 antibody. Cance Cell. 2022 In press.

15. Gfeller D, Guillaume P, Michaux J, Pak H-S, Daniel RT, Racle J, et al. The Length Distribution and Multiple Specificity of Naturally Presented HLA-I Ligands. J Immunol. United States; 2018;201:3705–16.

16. Zheng L, Ding D, Edil BH, Judkins C, Durham JN, Thomas DL 2nd, et al. Vaccine-Induced Intratumoral Lymphoid Aggregates Correlate with Survival Following Treatment with a Neoadjuvant and Adjuvant Vaccine in Patients with Resectable Pancreatic Adenocarcinoma. Clin Cancer Res. 2021;27:1278–86.

17. Lutz E, Yeo CJ, Lillemoe KD, Biedrzycki B, Kobrin B, Herman J, et al. A lethally irradiated allogeneic granulocyte-macrophage colony stimulating factor-secreting tumor vaccine for pancreatic adenocarcinoma. A Phase II trial of safety, efficacy, and immune activation. Ann Surg. 2011;253:328–35.

18. Hilmi M, Bartholin L, Neuzillet C. Immune therapies in pancreatic ductal adenocarcinoma: Where are we now? World J Gastroenterol. 2018;24:2137–51.
